# Editorial: Neurovascular dysfunction after brain injury

**DOI:** 10.3389/fphar.2023.1222328

**Published:** 2023-06-02

**Authors:** Chongjie Cheng, Lei Huang, Zongduo Guo, Tunan Chen

**Affiliations:** ^1^ The 1st Affiliated Hospital of Chongqing Medical University, Chongqing, China; ^2^ Loma Linda University Medical Center, Loma Linda, CA, United States; ^3^ Southwest Hospital, Army Medical University, Chongqing, China

**Keywords:** brain injury, vascular remodeling, vascular injury, neurovascular dysfunction, functional outcome

Till now, our understanding of neurovascular dysfunction in brain injuries was greatly expanded. Emerging experimental investigations suggested vascular failure is one of the key pathogenic factors, which essentially underlies the damaging secondary events, featured in early capillary permeability disruption leading to cerebral edema, inflammation, and eventual neuron death. At delayed phases, vascular remodeling, particularly in angiogenesis and enhanced endothelial-derived trophic factor secretion, may promote endogenous neurogenesis and neural restoration. Hence, novel strategies targeting early and delayed neurovascular dysfunction, might provide promising opportunities to improve clinical outcomes for patients following brain injuries. The goal of this Research Topic is to concentrate on the recent findings in the pathophysiology, diagnosis, and potential therapeutic targets of neurovascular changes following brain injuries.

In the Research Topic, three excellent reviews presented the advances in the pathophysiology and prognosis of neurovascular events following serial brain injuries. Luo et al. focused on the interaction between various components of the neurovascular unit and SUMOylation, and highlighted the importance of SUMOylation in neurovascular remodeling following acquired brain injury (ABI). Eastin et al. summarized the clinical findings of delayed revascularization in acute ischemic stroke patients. The existing evidence supports improved outcomes after subacute revascularization beyond 4.5 h of stroke onset in selected patients with favorable image characteristics. Chen et al. systematically reviews the pathophysiological changes of myelin sheath injury after Subarachnoid hemorrhage (SAH). They proposed that the precise prevention and treatment of myelin injury should focus on the spatiotemporal characteristics of myelin sheath changes and the initiation, intersection, and common action points of pathophysiological mechanisms, emphasizing the big picture other than details, and actively exploring different treatment methods.

Moreover, the original researches provided the therapeutic targets for potential medical translation. Chen et al. explored metabolomic alterations in mice after cerebral ischemia-reperfusion by employing metabolomic analysis coupling with GC-TOF-MS and UPLC/Q-TOF-MS. Their results showed several simultaneously differentially expressed metabolites and enriched pathways were significantly altered following ischemia-reperfusion. These varied metabolites are important for determining the cellular pathologic status of neuronal cells and their viability, and might be developed as potential molecular markers that can be helpful for therapeutic decision-making. Ma et al. explored the therapeutic effects of 9-phenanthrol after traumatic brain injury (TBI) in rats, and found that it hinders the expression and activity of MMP-9 by impeding TRPM4 channel and represses the destruction of BBB, thereby preventing vasogenic brain edema. Xu et al. performed a case-control study to investigate the correlation between DNA methylation and mRNA expression of the glutathione S-transferase alpha 4 (GSTA4) gene and the risk of intracranial aneurysm (IA) in the Chinese Han population. Their results suggested that GSTA4 methylation and mRNA expression may be reliable predictive markers of the occurrence of female IA.

Hopefully, the studies addressing our Research Topic will help understand the mechanisms underlying neurovascular dysfunction after brain injuries, and may provide promising targets to improve clinical outcomes in patients.

